# Meat and colorectal cancer in Middle Eastern and North African countries: update of literature review

**DOI:** 10.1186/s40985-020-00127-4

**Published:** 2020-05-11

**Authors:** Meimouna Mint Sidi Deoula, Khaoula El Kinany, Zineb Hatime, Hanae Abir Boudouaya, Karima El Rhazi

**Affiliations:** 1 Department of Epidemiology and Public Health, Faculty of Medicine and pharmacy of Fez, Sidi Mohamed Ben Abdellah University, Fez, Morocco; 2 Laboratory of Sciences Medical and Translational Research, Faculty of Sciences and Technology, Sidi Mohamed Ben Abdellah University, Fez, Morocco

**Keywords:** Colorectal cancer, Meat, Middle Eastern and North African countries, Prevention, Risk, Literature review

## Abstract

**Background:**

This review discusses the findings from epidemiological studies that have examined the possible role of meat and colorectal cancer (CRC) risk in Middle Eastern and North African (MENA) countries.

**Methods:**

We conducted a literature search in the PubMed, Clinical Trials, Google Scholar, Science Direct, and Cochrane databases for observational studies that investigated the association between meat and CRC risk in adults from the MENA region.

**Results:**

Eleven studies were included in this review. For red meat overall, significant associations were found. Regarding beef meat intake, the study included found controversial results with OR = 0.18 (95% CI 0.03–0.09). A positive association was observed between chicken and CRC risk, at OR = 2.52 (95% CI 1.33–4.77) to OR = 4.00 (95% CI 1.53–10.41) to OR = 15.32 (95% CI 3.28–71.45). A significant association was observed between processed meat intake and CRC risk, OR = 9.08 (95% CI 1.02–80.58).

**Conclusion:**

This is the first literature review which illustrated the association between meat consumption and CRC risk in MENA region. We concluded that these studies included in this review have been controversial and not sufficient to establish a clear relationship between CRC and meat consumption in the MENA region. Further studies are necessary to be carried out in this region, with a larger sample size and submit to rigorous criteria. This review will help researchers to improve the quality of future studies about the association between CRC and nutritional diet in general and meat in particular.

## Background

Colorectal cancer (CRC) is the third leading cause of cancer death and the fourth most commonly diagnosed cancer worldwide. In 2018, there were approximately 2 million new cases and 1 million deaths worldwide [1]. The incidence of CRC is higher in the developed countries compared with the developing countries [1]. Several studies have shown that there is a strong relationship between diet and the development of CRC [2, 3]. A large number of epidemiological studies have found a positive association between high intake of red meat and processed meat and CRC [4, 5]. In contrast, other studies have shown that there is no correlation between meat consumption and CRC risk [6]. Overall, most of these epidemiological studies have been conducted in developed countries, whose citizens adopt a Western diet rich in fat [7, 8]. In the other hand, a little information about this relationship in Middle Eastern and North African countries (MENA) is available. As compared to Western countries, the incidence of CRC in the MENA region is low, but it seems to have increased significantly during the last decade [9]. Moreover, the traditional diet in the MENA region is known to be healthy. This diet is characterized by a higher consumption of fruits, vegetables, and whole grains and lower to moderate in the consumption of meats and in the consumption of alcohol [10]. However, people from the MENA region are changing their traditional diet. A big part of this change is attributed to the globalization with the invasion of Western food rich in meat to the MENA countries [11]. In addition, this area has a many traditional foods of animal origin which, are widely consumed such as Gueddid, Pastirma, Khlii, Sujuk, Merguez, Tehal, Kourdass, and Nakanek [12, 13]. Moreover, they are mainly prepared at the household level under poor sanitary conditions [12]. The increase of CRC in this region probably is related to change of their traditional diet, in addition to these traditional meat products.

Consequently, the present review aimed at describing the associations between meat and CRC in Middle Eastern and North African countries.

## Methods

### Search strategy

We conducted an exhaustive search for full-text articles in databases: Pub Med, Clinical Trials, Google Scholar, Science Direct, and Cochrane databases, following the PRISMA guidelines [14], complemented by scrutinizing guidelines, databases, and references of identified publications. Search terms included fresh OR processed red meat OR white meat in combination with colon cancer OR rectal cancer OR colorectal cancer in MENA countries and by putting the combination of all these keywords. *Red meat* is mostly considered to be derived from mammals: beef, lamb, goat, veal, camel, pork, and rabbit. *White meat* is mostly derived from poultry, chicken, and turkey [15]. *Processed meat* is meat preserved by smoking, curing salting, or by the addition of chemical preservatives [16] used for a *cooking method* such as “steamed, grilled, tajine, roasted” types. *MENA countries* include Algeria, Bahrain, Egypt, Iraq, Iran, Israel, Jordan, Kuwait, Lebanon, Libya, Morocco, Oman, Palestine, Qatar, South Sudan, Sudan, Syria, Saudi Arabia, Turkey, Tunisia, the United Arab Emirates, and Yemen. All identified studies published until 31 October 2018 were considered.

### Eligibility criteria

The studies that were included in this review were original studies conducted among people living in the MENA region. All observational studies “prospective and retrospective” were held eligible for inclusion, only ecological and experimental studies were excluded. The studies that investigated the associations between meat consumption and CRC and provided estimates of the associations, by reporting the odds ratio (OR) or relative risk (RR) with 95% confidence intervals (CIs), were included. All the reviewed articles had been published in English or French.

### Quality assessment

Articles were selected independently by two investigators. Relevant publications were selected first upon reading of the title and abstract, and by reading the full text of the chosen articles. Several confounding factors (such as age, sex, tobacco and alcohol consumption) were considered in the selection procedure to ensure the questions validity. In addition, we determined the evidence level of all studies included in this review (Table 1).

**Table 1:** Quality assessment of published papers on meat and colorectal cancer in Middle East and North African countries

Author/Year/Reference	Relevant to this SR	Aims clearly stated	Appropriate study method	Sample representative of target population	Confounding and bias considered	Good response rate?	Were questions piloted?	Tables/figures understandable	Can results be applied to local situation?	Accepted as type IV evidence? [17]
Nashar and Almurshed, 2008 [18]	Yes	Yes	Yes	No	No	Yes	No	Yes	Yes	No (type III)
Bener et al., 2010 [19]	Yes	Yes	Yes	No	Yes	No	No	Yes	Yes	No (type III)
Guesmi et al., 2010 [20]	Yes	Yes	No	No	No	Yes	No	Yes	Yes	No (type III)
Arafa et al., 2011 [21]	Yes	Yes	Yes	No	Yes	No	No	Yes	Yes	No (type III)
Safari et al., 2013 [22]	Yes	Yes	Yes	No	Yes	Yes	Yes	Yes	Yes	No (type III)
Mahfouz et al., 2014 [23]	Yes	Yes	Yes	No	Yes	Yes	No	Yes	Yes	No (type III)
Abu Mweis et al., 2015 [24]	Yes	Yes	Yes	Yes	Yes	No	Yes	Yes	Yes	No (type III)
Tayyem et al., 2015 [25]	Yes	Yes	Yes	Yes	Yes	No	Yes	Yes	Yes	No (type III)
Azizi et al., 2015 [26]	Yes	Yes	Yes	No	Yes	Yes	Yes	Yes	Yes	No (type III)
Tayyem et al., 2016 [27]	Yes	Yes	Yes	No	Yes	Yes	Yes	Yes	Yes	No (type III)
El-Moselhy et al., 2017 [28]	Yes	Yes	Yes	No	Yes	Yes	No	Yes	Yes	No (type III)

## Results

**Fig. 1:**
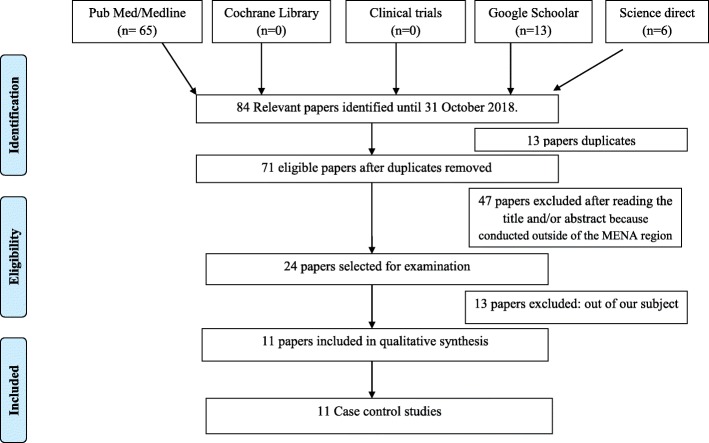
Flow diagram of the study inclusion of this literature review

The number of studies found until 31 October 2018 was 84. Among them, 72 papers were excluded (13 papers duplicates, 46 papers were conducted outside of the MENA region (Fig. 1) and 6 papers did not study the relation between meat intake and CRC risk and 8 papers did not precise the risk) [17, 29–41] (Table 2). Upon excluding the studies which did not meet the criteria (for the most part experimental studies), only eleven studies were singled out for reviewing (Fig. 1). The included studies represent six countries: Egypt, Jordan, Qatar, Saudi Arabia, Iran, and Tunisia. The methodological characteristics, the inclusion criteria of patients and the main exposures including the consumption of all types of meat and CRC risk have been summarized in (Table 3) as well as the strength of the findings represented by the study design (level evidence) [42], the methodological weaknesses, the biases, and the limitations of each study. The study results are summarized in Table 3 and described in the text.

**Table 2:** Characteristics of excluded studies

Author, date	Country	Type of study	Exclusion criteria
Rennert, 2007 [17]	Israel	Literature review	Did not study the relation between meat and CRC
Almurshed et al., 2009 [40]	Saudi Arabia	Case-control study	Did not study the relation between meat and CRC
Tayyem et al., 2013 [35]	Jordan	Case-control study	Did not study the relation between meat and CRC
Chenni et al., 2013 [37]	Algeria	Experimental study	Has been applied to animals
Rohani-Rasaf et al., 2013 [39]	Iran	Ecological study	Risk not specified
Aykan et al., 2015 [38]	Turkey	Cross-sectional study	Risk not specified
Ghahremani et al., 2016 [36]	Iran	Cross-sectional study	Did not study the relation between meat and CRC
Azzeh et al., 2017 [32]	Saudi Arabia	Case-control study	Risk not specified
Omran et al., 2017 [38]	Jordan	Cross-sectional study	Risk not specified
Al-Azri et al., 2019 [29]	Oman	Cross-sectional study	Risk not specified
Ilgaz and Gözüm, 2018 [30]	Turkey	Cross-sectional study	Risk not specified
Karimi et al., 2019 [31]	Iran	Cross-sectional study	Did not study the relation between meat and CRC
Mhaidat et al., 2018 [33]	Jordan	Cross-sectional study	Risk not specified
Nasaif and Qallaf, 2018 [34]	Bahrain	Cross-sectional study	Risk not specified

**Table 3:** Characteristics of included studies

Author/Year/Reference	Country and setting	Study design	Number of participants	Exposures and confounders	Outcome	Comparison	Main finding and effects
Nashar and Almurshed, 2008 [18]	Saudi Arabia in King Faisal Specialist Hospital and Research Center (KFSH&RC)	Case-control study	50 cases and 50 controls	Exposures: Dietary intake Confounders: Frequency of consumption	CC	Group I: 50 CRC cases were recruited in the KFSH&RC. Group II: 50 controls were selected in the same hospital of cases.	Lamb meat: OR = 13.5, 95% CI 2.64–68.84 Chicken with skin: OR = 4, 95% CI 1.53–10.41 Beef meat: OR = 0.18, 95% CI 0.03–0.090
Bener et al., 2010 [19]	Qatar in Al-Amal Hospital and Primary Health Care Centers	Case-control study	146 cases and 282 controls matched by age and gender	Exposures: Family history and lifestyle habits Confounders: BMI, smoking, family history, soft drinks, bakery products	CRC	Group I: 146 CRC cases were selected from the registered cases during the period January 2003 to December 2008. Group II: 282 controls were identified from the primary heath care centers as healthy with no history of any malignant tumor.	Frozen meat/chicken: OR = 1.20, 95% CI 0.77–1.87
Guesmi et al., 2010 [20]	Tunis in Charles Nicolle Hospital	Case-control study	32 cases and 61 controls	Exposures: Food group Confounders: Age, frequency of consumption (frequently/rarely), milk	CRC	Group I: 32 CRC cases were selected in the Charles Nicolle hospital. Group II: 31 patients with digestive pathology noncancerous were selected in the same hospital of the CRC cases. Group III: 30 patients with Traumatic pathology noncancerous were recruited from the same hospital of the other groups.	Delicatessen meat: OR = 5.1, 95% CI 1.4–18.5
Arafa et al., 2011 [21]	Jordan in Al-Bashir Hospital	Case-control study	220 cases and 220 controls matched by age and gender	Exposures: Dietary intake Confounders: Vegetables group, fruits, milk , yogurt, tea, bread	CRC	Group I: 220 CRC cases were recruited in Al-Bashir hospital. Group II: 220 controls were selected in the same hospital of cases.	Red meat: OR = 2.66, 95% CI 1.83–3.88 Saturated fat: OR = 1.03, 95% CI 1.01–1.05
Safari et al., 2013 [22]	Surgical units of the Cancer Institute of Imam Khomeini, Hospital Complex, and three major general hospitals (Shariati, Imam Hussein and Ayatollah Taleghani) in Tehran city, Iran	Case-control study	71 cases and 142 controls matched by age (within 5-year categories) and sex	Exposures: Dietary intake Confounders: Family history of CRC in first and second-degree relative, vegetable preparation, aspirin, acetaminophen, mineral and energy intake	CRC	Group 1: 71 cases with pathologically confirmed CRC, diagnosed no longer than six months before the interview, aged 40–75 years of age at the time of diagnosis and had no previous diagnosis of cancer at other sites, prior history of inflammatory bowel disease or familial adenomatous polyposis. Group 2: 142 controls were selected randomly from patients admitted to the same hospitals as cases during the same time period for acute, nonneoplastic conditions and not afflicted with diet-related chronic diseases.	“Western” dietary pattern (included sugar, processed and red meat, animal butter, refined cereals, tea, pickles, solid oil, mayonnaise, soft drink, legumes, sweets and desserts) increased the risk of CRC by OR = 2.616 (1.361–5.030) *p* = 0.004
Mahfouz et al., 2014 [23]	Egypt in El-Minia Oncology Centre	Case-control study	150 cases and 300 controls matched by age and sex	Exposures: Dietary intake Confounders: Alcohol intake, obesity, smoking, physical activity, alcohol, preserved food	CRC	Group I: 150 CRC cases were recruited in El-Minia Oncology Centre. Group II: 300 controls were selected in the same hospital of cases.	Red meat: OR = 57.1, 95% CI 12.1–270.3
Abu Mweis et al., 2015 [24]	Jordan in the King Hussein Cancer Center, King Abdullah University, Prince Hamzeh, Jordan University Hospital, and Al-Basheer Hospital	Case-control study	167 cases and 240 controls matched by age, sex, occupation, and marital status	Exposures: Food group Confounders: Age, sex, total energy intake, education level, marital status, work income, and family history	CRC	Group I: 167 CRC cases were recruited from the five major Jordanian hospitals, including an oncology center. Group II: 240 controls were selected randomly from among hospital personnel, out patients, visitors, and accompanying individuals.	Chicken: OR = 2.52, 95% CI 1.33–4.77 Red meat: OR = 0.64, 95% CI 0.37–1.11
Tayyem et al., 2015 [25]	Jordan in the King Hussein Cancer Center, King Abdullah University, Prince Hamzeh, Jordan University Hospital, and Al-Basheer Hospital	Case-control study	169 cases and 248 controls matched by age, sex, occupation, and marital status	Exposures: Macro-micronutrients consumption Confounders: Total energy intake, BMI , physical activity, family history, household income, marital status, and tobacco consumption	CRC	Group I: 169 CRC cases were recruited from five Jordanian hospitals specializing in oncology diagnosis and treatment. Group II: 248 controls were recruited from hospital personnel, outpatients, visitors.	Saturated fat: OR = 5.23 , 95% CI 2.33–11.76 Cholesterol: OR = 2.48, 95% CI 1.18–5.21
Azizi et al., 2015 [26]	Hospitals in Tabriz City of Iran	Case-control study	417 (207 cases and 207 controls) matched by age and sex (within 10-year categories)	Exposures: Dietary intake Confounders: History of diabetes, family history of CRC in first-degree relative, physical activity, BMI	CRC	Group 1: 207 cases with CRC (confirmed by pathology and colonoscopy findings, diagnosed no longer than 6 months before the interview). Group 2: 207 controls free of neoplastic conditions and diet-related chronic diseases (from the same hospital at the same period as the cases selected). Inclusion criteria were age 35–75 years old, CRC confirmed for the cases, being free of CRC for the controls and informed consent.	Significant association was observed between Iranian dietary pattern (included fried chicken, processed and red meat, black tea, carbonated beverage) and colorectal cancer after adjusting for history of CRC in first-degree relative, history of diabetes, and physical activity: OR = 1.46 (1.05–2.19), *p* = 0.021
Tayyem et al., 2016 [27]	Five large Jordanian hospitals with oncology services.	Case-control study	220 cases and 281 controls matched by age, sex, occupation, and marital status	Exposures: Meat, dairy products and fats Confounders: Age, sex, BMI, physical activity level, total energy intake, income, occupation, education level, marital status, cigarette smoking (current or lifelong; ever or never), other health problems and family history of CRC	CRC	Group I: 220 were diagnosed CRC cases were recruited conveniently from five large Jordanian hospitals with oncology services. Group II: 281 controls were recruited from hospital personnel, outpatients and visitors.	Chicken (OR = 15.32, 95% CI = 3.28–71.45, Ptrend = 0.009) and Mortadella, a type of processed meat (OR = 9.08, 95% CI = 1.02–80.58, Ptrend = 0.049) Steak: 0.42 (0.14–1.24) Liver: 2.88 (0.25–32.81)
El-Moselhy et al., 2017 [28]	General Surgery, Tropical Medicine, and Internal Medicine Clinics, Al-Azhar University Hospitals, Assiut and Cairo	Case-control study	160 cases and 300 controls	Exposures: Lifestyle, and socio-demographic and dietary data Confounders: BMI, physical activity	CRC	Group I: 160 patients with CRC attending the General Surgery, Tropical Medicine, and Internal Medicine Clinics, Al-Azhar University Hospitals, Assiut and Cairo. Group II: 300 healthy subjects (relatives to other patients attending these clinics and free from any type of cancer).	Processed meats intake (OR = 5.12, 95% CI 3.08–8.53) Low white meats intake (OR = 2.17, 95% CI 1.4–3.37) High animal fat intake (OR = 5.59, 95% CI 3.52–8.9)

Regarding red meat consumption, a positive association was observed with CRC risk in five case-controls studies, Jordan case-control studies conducted by Arafa et al. [21], two Iran case-control studies conducted by Safari et al. and Azizi et al. [22, 26], and Egypt [23] and Saudi Arabia [18], respectively (OR = 2.66, 95% CI 1.83–3.88; OR = 2.616, 95% CI = 1.361–5.030; OR = 1.46, 95% CI = 1.05–2.19; OR = 57.1 95% CI 12.1–270.3; OR = 13.5, 95% CI 2.64–68.84). Conversely, the case-control study conducted in Saudi Arabia by Nashar and Almurshed [18] has found an inverse association between beef meat intake and CRC risk with (OR = 0.18, 95% CI 0.03–0.90), whereas Abu Mweis et al. [24] from Jordan and Bener et al. from Qatar [19] have found no significant association between red meat intake and CRC risk, respectively (OR = 0.64, 95% CI 0.37–1.11; OR = 1.20, 95% CI 0.77–1.87).

Concerning the relation between processed meat and CRC risk, the three studies, from Egypt [23, 28], Tunisia [20], and Jordan [27], showed a positive association (OR = 2.4, 95% CI 1.5–3.8; OR = 5.12, 95% CI = 3.08–8.53; OR = 5.1, 95% CI 1.4–18.5; and OR = 9.08, 95% CI = 1.02–80.58, respectively).

For chicken, Nashar and Almurshed from Saudi Arabia [18] and Abu Mweis et al. [24] and Tayyem et al. from Jordan [27] showed a significant association between its consumption and CRC risk (OR = 4, 95% CI 1.53–10.41; OR = 2.52, 95% CI 1.33–4.77; and OR = 15.32, 95% CI = 3.28–71.45, respectively).

Regarding to the relation between saturated fat and CRC risk, the two Jordanian studies conducted by Arafa et al. and Tayyem et al. [21, 25] showed the significant association (OR = 1.03, 95% CI 1.01–1.05, OR = 5.23, 95% CI 2.33–11.76 respectively).

Finally, no studies have examined the relationship between traditional meat products in the MENA region and CRC risk.

## Discussion

The aim of this review was to describe the associations between meat and CRC risk in MENA countries. The results of this review showed that there were few studies conducted in this region, they did not cover all countries and did not include all types of meat, particularly traditional meat products.

All included studies have a low evidence level and results were not usually homogeneous. The relationship obtained between meat intake and CRC risk varies from one country to another, as it sometimes may vary in the same country. For instance, the case-control study conducted in Jordan by Arafa et al. [21] found a positive association between red meat intake and CRC risk, while another case-control study conducted by Abu Mweis et al. [24] in the same country reported no significant association. Another example is the case-control study conducted in Saudi Arabia [18] which showed a decreasing risk of CRC for beef meat consumption, while the case-control study conducted in Qatar [19] showed no significant associations between all types of meat and CRC risk.

Some results from this literature review [18, 21, 23, 24] were similar to those reported in a meta-analysis involving 19 prospective studies [43] and a large Japanese cohort study [44] and a large European cohort study EPIC [45]. Moreover, the result from the Jordanian study [24], which exhibited no significant association between red meat intake and CRC risk, was in agreement with a large meta-analysis [46]. On the other hand, some results were completely controversial between findings in this literature review and others outside MENA region studies. This was the case for three case-control studies [18, 19, 24] which reported a positive association between chicken intake and CRC risk. However, the results from a meta-analysis, which included 16 case-control studies and 5 cohort studies were completely controversial [47].

Furthermore, the study conducted in Saudi Arabia by Nashar and Almurshed [18] showed a positive association between lamb meat and CRC risk, and a negative association between beef meat and CRC risk, whereas a meta-analysis including 19 prospective cohort studies and comprising data from 15,183 CRC patients [48] found a positive association between beef and lamb consumption and CRC risk. In addition, a large cohort study conducted in Denmark and included 644 cases of colon cancer and 345 cases of rectal cancer found a positive association between lamb meat and colon cancer [49]. In fact, the beef consumption has a higher heme iron content (mean heme iron in cooked beef 2.63 ± 0.5 mg/100 g) compared to lamb consumption (mean heme iron in cooked lamb 1.68 ± 0.4 mg/100 g). One of the main hypotheses explaining the link between heme iron and CRC development is based on red meat pro-oxidative properties that could induce the oxidation of dietary polyunsaturated fatty acids [50]. Oxidation leads to the formation of lipid peroxidation and advanced glycation end-products, such as malondialdehyde or 4-hydroxynonenal, which are cytotoxic and genotoxic [50]. In addition, most of epidemiologic and experimental evidence support a major role of heme iron (abundant in red meat but far less in poultry), in the promotion of CRC risk especially by the consumption of red and processed meat [51].

Hence, we noted that the results found in Saudi Arabia by Nashar and Almurshed [18] about the relationship between beef consumption and CRC risk remain less logical than those found in the scientific research.

Finally, the studies included in this literature review have a number of limitations. All these studies have a low evidence level and took a small sample size, which is not representative of the target population. The included studies had a retrospective nature (case-control studies) and some limitations were presented in those retrospective studies such as biases related to memory, seasonal variations in fruits, vegetables, and plates and cooking techniques. Furthermore, the majority of studies did not exclude the participants that followed a diet such as diabetic and hypertensive patients and did not include the recently diagnosed patients (new cases), which may affect the quality of the collecting dietary data. The majority of studies used the FFQ (Food Frequency Questionnaire) which is susceptible to errors and choose one year to dietary recall time, which may not be sufficient to determine associations with a disease state that take years to be developed. On the other hand, some of studies did not adjust the consumption of meat with others exposure to determine the confounding factors such as body mass index, physical activity, and energy intake. This could perhaps explain such controversial results. Furthermore, most of case-control studies did not specify red meat types consumed; they reported only red meat consumption. In addition, most of case-control studies did not consider cooking methods for meat and its doneness levels.

The major strongest point of this review is that it is the first to summarize and evaluate the association of meat consumption and CRC risk in the MENA region. The main results were heterogeneous, not always the same as in the other countries and sometimes completely controversial. These findings have several limitations linked mainly to the design of the included studies which are susceptible to different forms of biases such as random error, misclassification, and confounding [52].

## Conclusion

These results are not only insufficient, but also unconvincing. Furthermore, no studies have worked on the traditional meat products in the MENA region, which may explain partly the increase of CRC risk in this region. Further studies are necessary to be carried out in this region, with a larger sample size and conducted in rigorous criteria. These findings will help researchers to improve the quality of future studies about the association between CRC risk and nutritional diet in general.

## Data Availability

All data available if you need you will contact the corresponding authors.

## Abbreviations

CRC: Colorectal cancer
MENA: Middle Eastern and North African
WHO: World Health Organization
FFQ: Food Frequency Questionnaire
